# FDNHSA’s response to KL Dunkle et al.’s ‘Upholding clinical integrity and South African relevance: A defence of the SASOP position statement on the care of transgender and non-binary youth’

**DOI:** 10.4102/sajpsychiatry.v32i0.2602

**Published:** 2026-01-27

**Authors:** Allan Donkin, Reitze Rodseth, Janet Giddy

**Affiliations:** 1 First Do No Harm Southern Africa, Cape Town, South Africa; 2 Department of Anesthesiology, School of Clinical Medicine, University of KwaZulu-Natal, Durban, South Africa

We value Dunkle et al.’s^1^ critique of our published call for the South African Society of Psychiatrists (SASOP) to review their position on the care of ‘transgender and non-binary youth’.^2^ Ongoing dialogue is crucial for safeguarding the best interests of South African children. Public trust in the medical profession is enhanced when criticism is addressed constructively.

Our original publication criticised SASOP for framing all children with gender dysphoria as being transgender, not following best practice in developing their statement, ignoring systematic reviews highlighting weak evidence for gender-affirming care (GAC), relying on circular references, overlooking alternatives to GAC, overlooking the harms of GAC, and omitting the limitations of informed consent for irreversible procedures in children.^2^ Dunkle et al.’s rebuttal does not adequately address these points but instead introduces new discussion points, including critiques of the Cass Review, global consensus arguments and the African context. We will briefly respond to each of these.

## Criticism of The Cass Review

The claim that our argument centres on the Cass Review’s policy recommendations for the UK is misleading. To clarify, our focus is on the global evidence base, which includes multiple systematic reviews,^3,4,5,6,7,8,9^ which is inadequate to support GAC for children – including in South Africa. Engagement with this evidence should be a prerequisite when developing a credible position statement on the care of transgender and non-binary youth.

The assertion that the Cass Review omitted affirming outcome data is incorrect. It evaluated 103 non-randomised observational studies using the Newcastle–Ottawa Scale; only two were of high quality, and 58% met sufficient standards for inclusion in the Cass Review analysis.^10,11^

Criticisms about its lack of accountability or transparency are unsubstantiated. Cheung et al.^12^ argue these critiques misrepresent both the process and the standards typical of United Kingdom (UK) independent reviews which are meant to minimise bias and conflict of interest, including that UK independent reviews are typically conducted by ‘a respected public figure with no ties to the area under review’.^11,12^ Kingdon et al.,^13^ McDeavitt et al.^14^ and Clayton et al.^15^ have responded to the common criticisms of the Cass Review, and have more than adequately defended its methodology. Engagement should focus on actual evidence, not advocacy group citations such as the Southern Poverty Law Center.^16^

Dunkle et al. state that most transgender youth benefit from access to GAC: ‘[the]overwhelming majority of transgender youth … benefit from access to care, or experience harm and distress when care is denied or made inaccessible’; but systematic reviews^3,4,5,6,7,8,9^ find no compelling evidence supporting this claim and consistently highlight the paucity of data on long-term outcomes. When a study purporting to prove evidence for the benefit of GAC is rated in a systematic review as being of low or very low certainty, then the true effect may be substantially different from the estimate of the effect or is likely to be substantially different from the estimate of the effect.^17^

Regarding the evidence base for an approach that focuses on psychological and social support rather than medicalisation – a thorough consideration of ethical principles recognises that when two approaches are associated with similar certainty of evidence, but one carries a lower risk of harm, a strong recommendation is supported in favour of the safer option.^11,18^

## Appeal to global ‘consensus’

Kazlowska et al. outline GAC treatment models ranging from rights-based to evidence-based approaches.^19^ We propose that consensus-based approaches are a third category. A claim to consensus by Dunkle et al., without strong evidence, risks repeating past medical system failures, such as frontal lobotomies, the thalidomide tragedy and the opioid crisis.

Dunkle et al. cite guidelines from French, German, Austrian and Swiss medical societies^20,21^ which, rather than being evidence-based, reflect consensus among clinicians with vested interests, including providing GAC to children, and who consequently have much to lose in an objective consideration of the evidence. WPATH, also consensus-driven, has been criticised for poor guideline quality,^22^ disregarding its own reviews and bowing to political interference.^11^ These are the sources underpinning the SASOP position statement.

Kulatunga-Moruzi et al.^11^ discuss how many countries have adopted an evidence-based approach including Finland,^23^ Sweden,^24^ the UK,^25^ Norway,^26^ Italy,^27^ the US^28^ and New Zealand.^29^ We suggest that SASOP do the same.

## African context

Contrary to claims about traditional African perspectives, child and adolescent GAC originates in and is largely driven by actors in the Global North, having emerged out of the Dutch protocol. Dunkle et al.’s references used in support of GAC are predominantly from the Global North,^16,20,21,30^ and GAC now represents a multi-billion-dollar industry, based on WPATH guidelines.^31^

## Conclusion

Dunkle et al. have not substantively addressed our critique of the SASOP position statement. Their new discussion points regarding evidence, consensus, and the African context sidestep valid critique. The authors do not produce robust evidence that convincingly shows GAC interventions to be safe, effective, and in children’s best interests.

Additional issues for SASOP to consider include the temporary nature of gender distress in many children and adolescents,^32^ the impact of social transition on concretising cross-gender identification,^33^ insights from desistance and detransition research,^34^ the role of psychiatric comorbidities,^35^ changes in demographics, particularly the increase in gender dysphoria in adolescent girls,^33^ and how the risks of puberty blockers and hormone therapies have been minimised.^11^

Clinicians entrusted with caring for children need to confront challenges and uncertainties objectively, using robust evidence. As doctors, we do not want to believe that we may be harming children, and this cognitive dissonance may evoke denial and rationalisation to avoid shame. It takes courage to admit that we may need to reconsider strongly held views and positions. This is our challenge to South African medical colleagues.
